# Good Quality of Life among People Living with Diabetes Mellitus Visiting the Outpatient Department of Endocrinology in a Tertiary Care Centre

**DOI:** 10.31729/jnma.8370

**Published:** 2023-12-31

**Authors:** Sumitra Aryal, Roshan Bhandari, Sabina Paudel, Radhika Khadka, Sita Adhikari, Maheshor Kaphle

**Affiliations:** 1 Department of Public Health, CiST College, New Baneshwor, Kathmandu, Nepal; 2 Department of Endocrinology, Tribhuvan University Teaching Hospital, Maharajgunj, Kathmandu, Nepal; 3 Department of Public Health, School of Health and Allied Sciences, Dungepatan, Kaski, Nepal; 4 Department of Public Health, Peoples Dental College and Hospital, Naya Bazar, Kathmandu, Nepal

**Keywords:** *diabetes mellitus*, *patients*, *quality of life*

## Abstract

**Introduction:**

Assessing the quality of life of individuals living with diabetes is crucial for ensuring optimal care and effective management of complications related to their condition. Diabetes is one of the leading causes of preventable mortality and morbidity among non-communicable diseases. The study aims to find out the prevalence of the good quality of life of people living with Diabetes mellitus visiting a tertiary care centre.

**Methods:**

A descriptive cross-sectional study was conducted among type 2 diabetic patients visiting the Outpatient Department of Endocrinology in a tertiary care centre from 30 June 2022 to 20 July 2022. Ethical approval was obtained from the Institutional Review Committee. A systematic random sampling technique was used. D-39 questionnaires were administered via face-to-face interviews. Point estimate was calculated at 95% Confidence Interval.

**Results:**

Among 118 patients living with diabetes, good quality of life was seen in 97 (82.20%) (75.3089.10, 95% Confidence Interval). This study found that the energy and mobility domain has the highest mean score of 26.7±7.8.

**Conclusions:**

The prevalence of good quality life of people living with Diabetes mellitus was found to be higher than other similar studies done in similar settings.

## INTRODUCTION

Quality of life (QoL) is deteriorating in diabetic patients as compared to non-diabetic patients, especially in the presence of complications.^1^ The physical, psychological, and social aspects of health that are impacted by a person's experiences, convictions, hopes, and expectations are referred to as their quality of life.^2^

Diabetes is one of the leading causes of preventable mortality and morbidity among non-communicable diseases.^3^ Diabetes mellitus (DM) is one of the most common metabolic disorders globally, which is characterized by inadequate insulin production by pancreatic cells.^4^ Globally, diabetes prevalence was 9.3% (463 million adults) in 2019.^5^ In the context of Nepal, the prevalence of diabetes was 8.4%.^6^

This study aims to determine the prevalence of good quality of life of diabetic patients visiting a tertiary care hospital.

## METHODS

A descriptive cross-sectional study was done among type 2 diabetic patients visiting the Outpatient Department of Endocrinology in Tribhuvan University Teaching Hospital, Maharajgunj from 30 June 2022 to 20 July 2022. Ethical approval was obtained from the Institutional Review Committee [Reference number: 537(6-11E2,078/079)] from the Institute of Medicine, Tribhuvan University, Maharajgunj, Kathmandu, Nepal. Patients above 18 years of age, and diagnosed with type 2 diabetes mellitus were included in this study. Patients with type 1 diabetes, gestational diabetes, and those who did not give consent for data collection were excluded from the study. Informed consent was taken from the respondent after clarifying the objectives of the research. A systematic random sampling method was used. The sample size was calculated by the formula:


$$\begin{array}{ll} \text{n} & ={\text{Z}}^{2}\times \frac{\text{p}\times \text{q}}{{\text{e}}^{\text{2}}}\\ & =1.96^{2}\times \frac{0.68\times 0.32}{0.09^{2}}\\ & =104 \end{array}$$

Where,

n = minimum required sample size Z= 1.96 at 95% Confidence Interval (CI)
p = prevalence of good quality of life taken as 68% from previous study^6^
q = 1-p
e = margin of error, 9%

The minimum required sample size was 104. However, 118 participants were included in our study. From the selected diabetic clinic, patient flow per day for 15 working days was 1035 during the study period. The sampling interval "k" was calculated using the following formula for systematic random sampling:

k = N/n

 = 1035/118

 = 8.77

Where,

k = sampling interval
N = estimated total population during the study period
n = minimum required sample size

The first participant was selected using the lottery method from the Outpatient Department register and the next ninth patient was selected till the minimum sample size was reached.

A face-to-face interview was used as a data collection technique and a semi-structured D-39 questionnaire tool was used. D-39 questionnaire tool was a standard tool that consisted of 39 items that are addressed in 5 domains, such as energy and mobility (15 items), diabetes control (12 items), social burden (5 items), anxiety and worry (4 items), sexual functioning (3 items). All items are administered using seven response categories, ranging from not affected at all (score= 1) to extremely affected (score= 7).^7^ Zero indicates the perfect quality of life and 100 indicates the low quality of life. The above 40 score was bad quality of life for the patient and the below 40 score, was good quality of life.^6^ Five domain was summed up, and the resulting raw scores were transformed into scales ranging from 0 to 100 using a linear transformation: (raw score-minimum value)/(maximum value-minimum value)× 100.^8^

Data were entered in Epidata 3.1 and analysed using IBM-SPSS version 20.0. The point estimate was calculated at a 95% CI.

## RESULTS

Among 118 patients living with diabetes, good quality of life was seen in 97 (82.20%) (75.30-89.10, 95% CI). Among these 97 respondents, the mean age was 54.62±10.76 years with 50 (51.50%) being female. In terms of religion, the majority were Hindu, accounting for 79 (81.44%) respondents, followed by 11 (11.34%) who identified as Buddhist. Only 11 (11.34%) of the respondents had no formal education. The occupation distribution indicated that 31 (31.95%) respondents were employed.

Amongst 97 who have a good quality of life, the domain of energy and mobility had the highest mean score of 23.57±5.23 (Table 1).

**Table 1:** Descriptive Statistics of Transformed Score of QoL (n= 97).

Domains	Mean
Energy and mobility	23.57±5.23
Diabetes control	22.62±4.17
Anxiety and worry	8.71±2.91
Social overload	6.58±1.32
Sexual behavior	3.80±1.35

There was a relatively high percentage of individuals with a good quality of life in each category. Among all 97 respondents, nearly the same percentage of respondents aged below and above 54 and both genders have good quality of life. Similarly, more than half of respondents whose education is secondary level have a good quality of life (Table 2).

**Table 2:** Sociodemographic status according to good quality of life (n= 97).

Variables	Good QoL n (%)
**Age (years)**	
<54	47 (48.45)
>54	50 (51.55)
**Gender**	
Male	47 (4S.45)
Female	50 (51.55)
**Education**	
Illiterate	13 (13.40)
<Secondary level	50 (51.55)
>Secondary level	34 (35.05)
**Occupation**	
Homemaker	30 (30.93)
Others occupation	67 (69.07)
**Religion**	
Hindu	79 (S1.44)
Non-Hindu	18 (1S.56)
**Ethnicity**	
Upper caste	41 (42.26)
Others	56 (57.74)

The results indicate that individuals who do not smoke 88 (90.73%) and do not consume alcohol 67 (69.07%) have a higher percentage of good quality of life compared to those who smoke and consume alcohol. Similarly, of individuals who engage in physical exercise 76 (78.35%) have a higher percentage of good quality of life compared to those who do not engage in physical exercise. Regarding medication cost, individuals with medication costs less than NPR 3000 have a higher percentage of 64 (65.97%) of good quality of life compared to those with medication costs more than 3000. Additionally, individuals with the presence of DM in the family have a lower percentage 37 (38.15) of good quality of life compared to those without DM in the family. Individuals on oral antihyperglycemic (OAH) treatment have a higher percentage 81 (83.5%) of good quality of life compared to those on insulin treatment (Table 3).

**Table 3:** Good Quality of life and health-related characteristics (n= 97).

Variables	Good QoL n (%)
Current smoker	9 (9.27)
Consume alcohol	30 (30.92)
Physical exercise	76 (78.35)
**Disease duration (years)**	
<10	59 (60.82)
>10	38 (39.18)
**Medication cost (NRP)**	
<3000	64 (65.97)
>3000	33 (34.03)
**The presence of other DM in the family**	**37 (38.15)**
**Treatment type**	
Insulin	3 (3.10)
Oral antihyperglycemic	81 (83.50)
Both	13 (13.40)

## DISCUSSION

This study was conducted to assess the quality of life of people living with diabetes mellitus in Kathmandu metropolitan city by using the D-39 questionnaire. This study found that 97 (82.20%) of respondents had good quality of life. A similar cross-sectional study has shown 69% good quality of life.^6^ Another study conducted in India has found that 68% of participants have a good quality of life.^9^ This slight variation was due to study area, sample size, and way of analysis.

The highest mean of 23.57±5.23 was found to be in the energy and mobility domain, followed by diabetes control with a mean score of 22.62±4.17. A similar study conducted in eastern rural Nepal has found that the social burden domain has the highest mean score of quality of life.^10^ These two results contradicted each other which may be due to the urban areas and rural areas. Sexual functioning is the least affected quality of life and this finding was also supported by the study conducted in Pakistan.^11^ One of the studies has found that the diabetes control domain is least affected.^12^ The study conducted in Pokhara metropolitan city found that anxiety and worry had the highest mean score of quality of life. A study revealed that the energy and mobility domain and diabetes control domain have the highest mean scores, indicating that these domains have a lower impact on the quality of life of diabetic patients. A study found that there is a difference in the quality of life of individuals between those with disease duration but different studies have revealed that a longer duration of diabetes mellitus leads to worsening the quality of life.^13^ These findings were contradicted by another research.^14^ It might be a change in the attitude of the participants towards the treatment regimen. This study found that individuals with the presence of complications have a lower percentage of good quality of life compared to those without complications. This finding is also supported by the research conducted in the USA on adults and various other studies which showed the presence of complications have a poor quality of life in a diabetic patient.^15^ Among the presence of complications of diabetes, 57.1% of the respondents suffered from hypertension; the most common complication. One of the studies done in a tertiary care diabetes centre in Karachi-Pakistan found that 57.2% of the participants were hypertensive.^11^

This was a hospital-based study so the findings may not be generalizable to a community setting.

## CONCLUSIONS

The prevalence of high quality of life among patients living with diabetes mellitus was higher than other studies done in similar settings.
